# Discontinuation, switching and contraceptive failure patterns of long-acting reversible contraceptive users in Kenya: a quantitative study

**DOI:** 10.1080/26410397.2025.2603740

**Published:** 2026-01-09

**Authors:** Midanna de Almada

**Affiliations:** PhD Candidate, Department of Methodology, London School of Economics, London, UK.

**Keywords:** long-acting reversible contraceptives, contraceptive dynamics, contraceptive failure, contraceptive discontinuation, pregnancy outcomes

## Abstract

The number of IUD and implant (long-acting reversible contraceptive) users in sub-Saharan Africa is increasing. Yet, little is known about LARC users’ experiences and their discontinuation, switching, and failure rates. Contraceptive behaviours can support our understanding of users’ experiences and whether these methods meet their needs; this understanding can inform user-centred contraceptive programming and decision-making. This study applies event history analysis and life tables to the Kenyan Demographic and Health Survey contraceptive calendar data to examine LARC users’ behaviours and patterns compared to users of other methods. It also measures pregnancy outcomes following contraceptive failure by the method that failed. Results show that contraceptive failure may be under-estimated due to an underreporting or misreporting of failure. Alongside self-reported failure, this analysis uses a derived failure code, which finds that LARC typical-use failure rates may be higher than reported in other studies. LARC users are less likely to switch or discontinue methods compared to other contraceptive method users. However, early LARC discontinuation due to a desire to conceive increases with duration of use. Additionally, LARC users are more likely to experience pregnancy termination following method failure than users of other contraceptive methods. This study has implications for bodily and contraceptive autonomy, contraceptive user’s experiences, user-centred contraceptive counselling, and the provision of maternal health and abortion services.

## Plain language summary

Contraceptive users’ behaviour can highlight whether the methods they use meet their needs and allow them to achieve their reproductive goals. This study examines: (1) How often and how many users stop a method; (2) How long have they used it; (3) Whether they switch to another method and (4) If they experience contraceptive failure (becoming pregnant while using contraception). The study includes all contraceptive methods but has a specific focus on the IUD and implant. These are becoming increasingly popular in sub-Saharan Africa. Despite this increase, little is known about users’ experiences with these methods. Using quantitative methods, this research finds that contraceptive failure may be underreported, and the IUD and implant may fail more often than previously believed. IUD and implant users are also less likely to switch to another method or stop use compared to users of other methods. Following a contraceptive failure, IUD and implant users are more likely to experience a pregnancy termination compared to individuals whose method failed but were not using the IUD or implant. By analysing these patterns, this study aims to centre bodily and reproductive autonomy, improve contraceptive counselling, ensure methods better align with users’ preferences and support the healthcare system in anticipating demand for healthcare services.

## Résumé

Le nombre d’utilisatrices de dispositifs intra-utérins (DIU) et d’implants (contraceptifs réversibles à longue durée d’action) est en augmentation en Afrique sub-saharienne. Pourtant, on sait peu de choses sur leurs expériences, notamment sur les taux d’arrêt, de changement et d’échec de ces méthodes. L’analyse des comportements contraceptifs peut nous aider à comprendre l’expérience des utilisatrices et à déterminer si ces méthodes répondent à leurs besoins. Cette compréhension peut guider la programmation et la prise de décision en matière de contraception axée sur l’utilisatrice. Cette étude applique l’analyse de l’historique des événements et les tables de mortalité aux données du calendrier contraceptif de l’Enquête démographique et de santé du Kenya afin d’examiner les comportements et les habitudes des utilisatrices de contraceptifs réversibles à longue durée d’action, comparativement aux utilisatrices d’autres méthodes. Elle mesure également les grossesses survenues après un échec de la contraception, en fonction de la méthode ayant échoué. Les résultats montrent que l’échec de la contraception pourrait être sous-estimé en raison d'une sous-déclaration ou d'une déclaration erronée de cet échec. En même temps que les échecs autodéclarés, cette analyse utilise un code d’échec dérivé qui révèle que les taux d’échec des contraceptifs réversibles à longue durée d’action d’usage courant pourraient être plus élevés que ceux qui sont rapportés dans d’autres études. Les utilisatrices de contraceptifs réversibles à longue durée d’action sont moins susceptibles de changer de méthode ou d’interrompre la contraception que les utilisatrices d’autres méthodes contraceptives. Néanmoins, l’arrêt précoce de ces contraceptifs réversibles à longue durée d’action en raison d’un désir de grossesse augmente avec la durée d’utilisation. De plus, les utilisatrices de contraceptifs réversibles à longue durée d’action risquent davantage de subir une interruption de grossesse après un échec de la méthode que les utilisatrices d’autres méthodes contraceptives. Cette étude a des conséquences pour l’autonomie physique et contraceptive, les expériences des utilisatrices, le conseil en matière de contraception centré sur l’utilisatrice et les services de santé maternelle et d’avortement.

## Resumen

El número de usuarias de DIU e implantes (métodos anticonceptivos reversibles de acción prolongada, MARAC) en África subsahariana está aumentando. Sin embargo, no se sabe mucho sobre las experiencias de las usuarias de MARAC y sus tasas de abandono, cambio y falla. Los comportamientos anticonceptivos pueden ayudarnos a entender las experiencias de las usuarias y saber si estos métodos satisfacen sus necesidades; esta comprensión puede informar los programas y la toma de decisiones sobre anticoncepción centrados en las usuarias. Este estudio aplica el análisis de la historia de acontecimientos y tablas de vida a los datos del calendario de métodos de la Encuesta Demográfica y de Salud de Kenia para examinar los comportamientos y patrones de las usuarias de MARAC comparadas con usuarias de otros métodos. Además, mide los resultados de embarazo después de la falla anticonceptiva por método que falló. Los resultados muestran que la falla anticonceptiva podría subestimarse debido al subregistro o a la notificación errónea de fallas. Además de la falla auto informada, este análisis utiliza un código de falla derivado que determina que las tasas de falla de uso típico de MARAC podrían ser más altas que aquellas informadas en otros estudios. Las usuarias de MARAC son menos propensas a cambiar de método o abandonar su método, comparadas con otras usuarias de métodos anticonceptivos. Sin embargo, el abandono temprano de MARAC debido al deseo de concebir aumenta con la duración de uso. Además, las usuarias de MARAC son más propensas a experimentar una interrupción del embarazo después de la falla del método que las usuarias de otros métodos anticonceptivos. Este estudio tiene implicaciones para la autonomía corporal y anticonceptiva, las experiencias de las usuarias de anticonceptivos, la consejería anticonceptiva centrada en la usuaria y la prestación de servicios de salud materna y aborto.

## Introduction

### Background

Long-acting reversible contraceptives (LARCs) – the intrauterine device (IUD) and subdermal implant – have been hailed by policymakers, global health researchers and family planning practitioners for their efficacy in preventing unintended pregnancy. With a < 1% clinical failure rate, LARCs eliminate much of the human error. Furthermore, they are more cost-effective when used for their full duration, and they provide efficacy for longer durations than their short-acting counterparts. Governments, donors and clinicians have made efforts to increase LARC use to support the realisation of demographic and health goals. However, critical examination of the experiences of LARCs, including in African countries, is underexamined.

Initiatives like Family Planning 2020 and 2030 (FP2020/ 2030)* have included commitments from donors, implementing partners and governments to implement LARC promotion and education, create demand, increase human resources for LARC counselling and referrals, and set targets for LARCs as a percentage of the contraceptive method mix.^1^ Through multi-donor initiatives such as the Implant Access Programme (IAP), LARCs are becoming more affordable and accessible for governments in the Global Majority countries.† Under IAP, the annual procurement of implants in the 69 FP2020 focus countries increased tenfold, from 1.7 million units in 2010 to 10.8 million units in 2018, without evidence of overstocking.^2^

These commitments and efforts are reflected in increasing proportions of LARC users in many African countries. In some countries, the proportion of implant users increased sixfold in less than a decade; in Kenya, LARC methods went from 2.3% (IUD) and 7.1% (implant) in 2014 to 4% (IUD) and 19% (implant) in 2022.^3,4^ In Burkina Faso in 2010, the IUD and implant were 0% and 3%; by 2021, they rose to 2% and 16%, respectively.^5,6^ The IUD and implant increased to 2% and 27% (2019/20) versus 0.4% and 4.7% in 2014/15 in Rwanda.^7,8^

### LARC literature

There is a growing body of literature from Global Minority countries that highlights concerns about LARCs. A troubling finding identified in Global Minority countries is a “method first” approach to contraceptive care, where individuals are educated primarily on LARCs but receive little to no information on other methods.^9,10^ This reduces an individual’s autonomy to make an informed choice about whether to use contraception and, if so, which method. Other studies reveal coercion and biases related to the provision of LARCs. Low-income individuals, people of colour, those with physical or intellectual disabilities, those who have had an abortion, drug and alcohol users, incarcerated individuals, and individuals whose children have been placed in care by the state were found to be more likely to be given or pressured into accepting a LARC.^10–12^ MuItiple vulnerabilities may make individuals even more likely to be coerced.

Studies highlight the role of provider bias and institutional racism/discrimination in LARC counselling and provision. Providers may have assumptions or unconscious biases about the need, appropriateness, and/or benefit of a LARC for an individual, depending on their characteristics and identities, influencing contraceptive counselling and, in turn, pressuring and/or coercing an individual into using a LARC. Morison^13^ found that LARC providers in New Zealand justify the use of coercive practices in the name of acting in the patient’s best interest. United States (US) and UK evidence show that some individuals are denied removal when choosing to discontinue LARC use.^12,14^ Mann et al^15^ found that some US women felt they were coerced into a postpartum LARC and, when seeking removal, had to “make a convincing case” to their provider. A qualitative study from the UK found that 43.8% of LARC users felt pressured into using a LARC, and more than half (56%) faced difficulties getting their LARC removed.^12^ Research highlights the focus on LARC insertion without ensuring the availability of removal services, particularly in low-resource settings.^16^ While some literature focuses on explicit forms of reproductive control, such as forced sterilisation, these findings regarding LARCs highlight more subtle and covert, but equally troubling, forms of reproductive coercion which have been referred to as “soft sterilisation”.^17–19^

However, much less is known about LARC users’ experiences in Global Majority countries, including in Africa. In an anonymised African country, Senderowicz^20^ found indications of contraceptive coercion. This included a target-driven approach that created a competitive environment among healthcare workers, biased and/or directive counselling, a lack of other contraceptive methods being offered and difficulty in having a LARC removed. Some women were given LARCs without their knowledge and/or consent. Respondents in this study reported trust in the motives and tactics of healthcare workers, even if they were coercive, believing they were acting in the patient’s best interest.

A study of non-preferred method use in Burkina Faso^21^ found that 31% of IUD users reported that it was not the method they originally wanted. 30% of implant users reported they would like a different method. In Tanzania, providers were less likely to counsel postpartum women on methods other than the IUD.^22^ While unsuccessful implant discontinuation declined between 2016 and 2020 in Burkina Faso (7% to 3%) and Kenya (9% to 2%), a substantial number of respondents were still unsuccessful in having their method removed.^23^ The main barriers to removal were being persuaded against discontinuation and being told to return another day.

### Study scope and rationale

Evidence highlights a potential infringement on an individual’s bodily and contraceptive autonomy. Bodily autonomy refers to one’s right to make decisions about one’s own body, free from constraints, violence and coercion, such as when to marry, have sex and/or become pregnant. Bodily autonomy is fundamentally connected to power, agency, freedom, choice and dignity.^24^ Closely related to bodily autonomy is contraceptive autonomy, defined as “the factors that need to be in place in order for a person to decide for themselves what they want in regard to contraceptive use, and then to realize that decision”.^25^ It includes three subdomains: informed choice, full choice and free choice. Drawing on frameworks of bodily autonomy and contraceptive autonomy, this paper uses a case study of Kenya to address knowledge gaps in our understanding of LARC users’ experiences. In lieu of primary data on user satisfaction and experiences, it measures LARC users’ discontinuation, failure and switching patterns compared to users of other contraceptive methods to highlight an aspect of user experience. This analysis moves beyond measuring contraceptive dynamics as a driver of unintended pregnancy and fertility rates to instead focus on what contraceptive dynamics might tell us about users’ experiences. As the number of LARC users increases and is emphasised in policies, it is important to understand if LARCs meet users’ needs and allow them to achieve their reproductive goals. This paper expands on recent work to understand if the troubling findings reported elsewhere might be present in Kenya. At the core of this work is the individual contraceptive user; how users behave is one dimension that can help inform these questions and considerations.

Kenya was chosen as a case study for four reasons (i) data recency (2022), (ii) the increase of LARC users in the past decade, (iii) its inclusion as an FP2030 commitment maker (i.e. the government has made specific commitments as part of FP2030) and (iv) Kenya’s FP2020 commitment to “broaden access and choice […] through the provision of long-acting and permanent methods of family planning. The Government will also scale up its efforts to equip health providers with skills on provision of long-acting methods … ”.^26^

This paper analyses three outcomes: contraceptive failure, discontinuation and switching. Contraceptive failure can result in (presumed) unintended/ unwanted/ mistimed pregnancies. Understanding typical-use LARC efficacy in different contexts can increase our understanding of the likelihood of unintended pregnancy, more accurately inform family planning programming and counselling and improve estimations of anticipated demand for health services, including maternal health programming and the provision of safe abortion services. Where contraceptive failures end in induced or spontaneous abortion or unwanted/mistimed birth, this analysis measures whether pregnancy outcome differs depending on the method that failed, drawing on Curtis and Blanc’s^27^ conceptual model. If contraceptives, specifically LARCs, fail more frequently than suggested by clinical trials or other studies, this has implications for the health and well-being of users, as well as for the services designed to support people experiencing unintended, unwanted or mistimed pregnancies.

Meeting user needs includes the success of a method in preventing pregnancy and the continuity of use. (Dis)continuation is an indicator of the success and quality of contraceptive methods and programmes.^28^ This research examines how long individuals use a method before discontinuing and/or switching and the reasons given for discontinuation. Patterns of early LARC discontinuation can highlight dissatisfaction, negative side effects, potential coercion, or perhaps a lack of knowledge regarding their long-acting nature. A recent meta-analysis also highlighted how a method’s influence on an individual’s sex life can be a reason for discontinuation and/or switching.^29^ What methods – if any – LARC users switch to when compared to users of short-acting methods may also inform knowledge and preference for long-acting/ provider-initiated methods versus short-acting/ self-initiated methods. Variation in reasons for discontinuation by individual method can highlight method-specific concerns. For example, if cost is reported as a reason for injectable discontinuation compared to other methods, this highlights a need for specific policies to address it. Disaggregating contraceptive dynamics by sociodemographic characteristics can highlight variability across populations, providing an understanding of which groups may be at risk of failure, discontinuation and/or switching.

### Contraceptive dynamics and pregnancy outcomes

Around 30% of unintended pregnancies are estimated to be the result of contraceptive failure.^30^ Perfect-use contraceptive effectiveness measures how effective a method is when used consistently and correctly, whereas typical-use failure reflects the real-life failure of a method when incorrect use, user action, or other external interferences are accounted for. LARC-specific studies that have used clinical data or conducted a literature review of studies that used clinical data (Table 1) tend to report a less than 1% failure rate for LARCs.

**Table 1.** Failure/efficacy rates and continuation rates of LARCs using clinical data

Failure rates from selected clinical studies					
First author (Year)	Location	LARC method	Follow-up period	Failure/efficacy rate	Continuation rate
Agrawal & Robinson^31^	Luton, UK	Etonogestrel subdermal implant	3 years	100% efficacy rate	30.2%
Aisien^32^	Benin City, Nigeria	Etonogestrel subdermal implant	5 years	100% efficacy rate	58.7%
Aisien & Enosolease^33^	Nigeria	Etonogestrel subdermal implant	12 months	100% efficacy rate	93.8%
Arribas-Mir et al.^34^	Granada, Spain	Etonogestrel subdermal implant	2 years, 9 months	100% efficacy rate	75%
Backman et al.^35^	Finland	Levonorgestrel IUD	5 years	Cumulative pregnancy rate was 0.5 per 100 users	Not reported
Díaz et al.^36^	Campinas, Brazil	Levonorgestrel IUD,	7 years	Zero pregnancies in 899 women-years	49.5% (after 3 years) and 23.7% (after 7 years)
Eroglu et al.^37^	Ankara, Turkey	Immediate postplacental, early postpartum, and interval (more than 6 weeks) IUD insertions	12 months	3.1% failure rate for all IUD types	80.7% (immediate postplacental); 63.6% (early postpartum), 90.4% (interval)
Gezginc et al.^38^	Turkey	Etonogestrel subdermal implant	2 years	100% efficacy rate	54%
Gupta et al.^39^	Papua New Guineau	Etonogestrel subdermal implant	12 months	0.8% failure rate	97%
Iftikhar et al.^40^	Pakistan	Etonogestrel subdermal implant	3 years	100% efficacy rate	73%
Lopez Del Cerro et al.^41^	Spain	Etonogestrel and levonorgestrel implants	Retrospective study spanning 9 years (2006–2015)	0.0 pregnancies per 100 women – years	52.5%
Mansour et al.^42^	N/A (Literature review)	Implant; levonorgestrel IUD	12 months	0–0.6 per 100 at 12 months (implant); 0.1–1.5 per 100 at 12 months (Cu-IUDs).	N/A (not reported)
Meirik et al.^43^	Multi-country	Levonorgestrel implant, IUD, and sterilization	5 years	Less than 1 per 100 woman-years failure rate for each method	66.8% (levonorgestrel implant); 69.5% (copper IUD); 73% (Non-copper IUD)
Stoddard et al.^44^	N/A	Levonorgestrel IUD	N/A	First-year failure rate of 0.2%	N/A
Thamkhantho et al.^45^	Bangkok, Thailand	Etonogestrel subdermal implant	12 months	100% efficacy rate	65.1%

While clinical studies tend to use clinical data and medical records to assess contraceptive effectiveness, social science research relies more on survey data, which tend to report higher failure rates. In their study of 43 low- and middle-income countries using DHS calendar data, Polis et al^30^ observed variation in the failure rate by countries/regions with the implant ranging from 0.2 to 1.3 per 100 episodes of use and 0.9–2.2 per 100 episodes of use for the IUD. Some of these estimates were higher than those found for the US, with 0.05% for the implant, 0.8% for the copper IUD, and 0.1% for the levonorgestrel IUD within the first year of use.^46^ Using US survey data from 2006 to 2010, the 12-month probability of failure for LARCs was 1%.^47^ Using survey data in France, the cumulative percentage of women in 2000 experiencing a failure using the IUD was 1.1% at 12 months, 4.2% at 24 months, 4.7% at 36 months and 5.2% at 48 months.^48^ Cleland & Ali^49^ measured contraceptive failure in 19 developing countries using DHS survey data. The 12-month failure rate for the countries where the IUD was included ranged from 0.7 per 100 episodes in Peru (2000) to 4.3 per 100 episodes in Colombia (2000), and the average failure rate across all countries was 1.7 per 100 episodes. Although Kenya was included in the Polis et al^30^ study, the data were from 2003, when neither the IUD nor the implant was recorded in calendar data.

There is a gap in the literature about whether pregnancy outcome varies depending on the method that failed. In Denmark, half (52%) of the contraceptive failures that resulted in unintended pregnancy were terminated by an induced abortion, and most of these unintended pregnancies were condom (59%) and pill (30%) failures.^50^ Only one study was found that assessed the pregnancy outcome of contraceptive failure in the Global Majority. Cleland & Ali^49^ found that 75.7% of the pregnancies that resulted from a contraceptive failure were carried to term, and just over one tenth ended in abortion, miscarriage or stillbirth. It is important to highlight and consider the role that the legality of and access to (safe) abortion services have in different settings and how pregnancy outcomes following a contraceptive failure will differ by setting based on these factors.

## Data and methods

This paper uses contraceptive calendar data from the Kenya 2022 DHS, a nationally representative household survey. DHS calendar data includes monthly calendars with information on pregnancies, births, terminations (DHS aggregates miscarriages, abortions and stillbirths as terminations) and episodes of contraceptive use, including the method being used and self-reported reason for discontinuation, for the previous five years (2017–2022). This paper uses both multiple-decrement life tables and discrete-time event history analysis to study contraceptive dynamics. Multiple-decrement life tables offer a descriptive analysis of the data, and event history analysis (EHA) estimates the effects of covariates on failure, discontinuation and switching rates. Multiple-decrement life tables measure the cumulative proportion of contraceptive episodes that are discontinued, fail or are switched from. EHA measures the hazard of failure, discontinuation and switching. Contraceptive dynamics are measured at 12, 24, 36 and 48 months; because this paper focuses on LARCs, longer time periods are analysed to capture the long-term use of these methods and experiences of failure, discontinuation and switching. As a secondary analysis of pre-existing publicly available data, ethics committee approval and informed consent were not required for this study.

DHS definitions of failure and discontinuation are used in this analysis.^51^ Failure is defined as an episode of contraceptive use that failed, or resulted in a pregnancy, in the month directly following a month of contraceptive use. Discontinuation is defined as an episode of contraceptive use that ends for any reported reason. Switching is defined as an episode of contraceptive use that ended but is followed within the next two months by another method of contraception. If an individual discontinued a method and then resumed the same method, they are categorised as switching.

The approach to analysis considered how to define and measure switching. Switching was considered to be a user who discontinued a method and initiated a new method within two months. There is a lack of standardisation in the definition of switching within contraceptive research; studies have considered switching within one month,^52^ two months,^53^ three months^54^ or baseline versus 12-month follow-up.^55,56^ A within two-month initiation of a new method was selected to account for delays or constraints that might prevent method initiation within one month, while also considering risk for an unintended/ mistimed/ unwanted pregnancy during non-use.

### Life table methods

**Figure 1.**
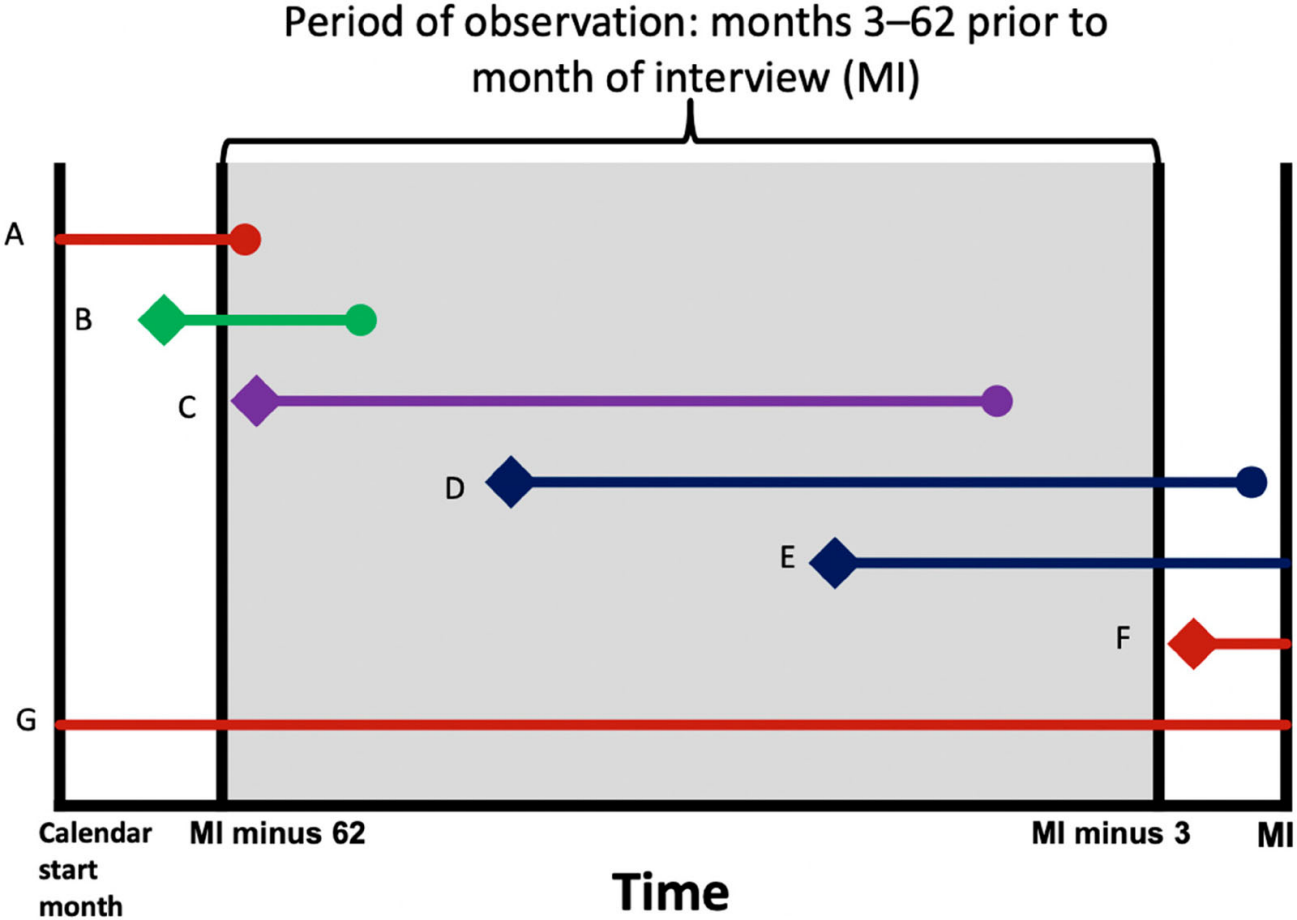
Graphic from Polis et al.^30^ showing full inclusion (purple), exclusion (red), late entry (green) and censoring (blue) for calendar observations

The period of observation for the contraceptive calendars is months 3–62 before the month of the interview. This is done to standardise the calendar length for all women since the month of the interview varies for respondents; hence, the length of the calendar for each woman varies depending on the month in which they were interviewed.^30^ It also accounts for women in the first trimester of their pregnancy who may not yet know they are pregnant. Including these months could lead to an underestimation of failure, as their contraceptive use would be included, but not the contraceptive failure.^30^ In the current analyses, the data were right-censored for incomplete observations and left-truncated; only episodes that began within the calendar period and ended three months before the interview are fully included in the life tables. Episodes that began before the start of the calendar are excluded as their start date could not be determined, and individuals who used the same method for the duration of the calendar (i.e. start and end date unknown) are also excluded. Late entries occur when an individual starts a method within the calendar period but before the observation period (3–62 months). They are included as a late entry, where only the months of contraceptive use that occurred within the observation period are included.^30^ Figure 1^30^ shows inclusion and exclusion where A, F and G are excluded, B is treated as a late entry, and D and E are included but censored 3 months before the month of interview.

The life tables include self-reported reasons for method discontinuation. The DHS provides 15 method discontinuation codes. For ease of analysis and interpretation, these codes were grouped. Groupings were based on similarities among the reasons specified. Some reasons of interest were kept separate, such as failure or desire for a more effective method. Sample sizes were also considered when grouping, as some options had a comparatively small representation, such as fatalistic (0.13% of discontinuation reasons), cost (0.64% of discontinuation reasons) and marital dissolution (1.1% of discontinuation reasons). The 15 codes are grouped into eight categories:

- Method failure
- Desire to become pregnant
- Fertility and marital-related reasons (infrequent sex/ husband away; husband disapproved; fatalistic; difficult to get pregnant, marital dissolution)
- Side effects/ health concerns
- Wanted more effective method
- Method and accessibility barriers (lack of access/ too far; inconvenient to use; cost; access/ availability)
- Other/ Don’t know
- Switching

### Event history analysis methods

Calendar data for the discrete-time EHA were reshaped into person-month format, where each row represents one month of calendar data. The data were right-censored and left-truncated. Separate logistic regression models were fitted to estimate the monthly probability of each event (failure, discontinuation, switching) at 12, 24, 36 and 48-month intervals. In each model, time was measured in months since method initiation and included as a covariate for variation in the baseline hazard over time. Odds ratios from these models approximate discrete-time hazard ratios. The model is:

$$\mathrm{logit}\;(P(Y_{\mathrm{it}}=1\;|X_{\mathrm{it}}))=\alpha +\;\beta_{1}X_{1\mathrm{it}}+\;\beta_{2}X_{2\mathrm{it}}\ldots \;\beta_{k}X_{\mathrm{kit}}$$

where $Y_{\mathrm{it}}$ is the binary outcome indicating whether the event occurs for individual i at time t, $P(Y_{\mathrm{it}}=1\;|X_{\mathrm{it}})\;$ is the probability of an event occurring for individual i at time t for those whom the event has not yet occurred for time t–1, α is the intercept, And $\beta_{1},\;\;\beta_{2\;}$ … . are the coefficients of covariates $X_{1\mathrm{it}},\;\;\beta_{2}X_{2\mathrm{it}}$ … .

To measure failure and discontinuation in EHA, contraceptive methods were grouped. For failure analysis they were grouped using an approach based on Festin et al’s typology^57^ into LARCs (implant and IUD), more effective short-acting methods (pill, injectable), less effective short-acting methods (condom, emergency contraception, standard days, other modern methods) and least effective methods (withdrawal, periodic abstinence/ rhythm, other traditional methods). To measure discontinuation, these methods were grouped into self-discontinued (i.e. a method someone could independently discontinue) and provider-discontinued (i.e. a method requiring a trained clinician for removal). All methods except the IUD and implant are categorised as self-discontinued. Women who were sterilised were removed from all analyses because there were no cases of sterilisation failure in the sample and because discontinuation or switching are not possible after tubal ligation. Lactational amenorrhoea was excluded since it is a method that is recommended to be used for a finite period of time.

The ways in which a contraceptive spell can end in EHA are treated as competing risks and can “end” either as (1) continuing to use the same method, (2) failure, (3) discontinuation or (4) switching (which is also discontinuation of the original method). Each woman in the sample can experience multiple episodes of contraceptive use throughout the calendar period, meaning that episodes are nested within one individual, and there may be repeated observations. However, since each episode or event can only have a single ending (i.e. continuation, discontinuation, switching, or failure), each event is censored and treated independently. To avoid underestimating the standard error and account for the clustered nature of the data, a clustered standard error was used in the EHA regressions.

### Pregnancy outcomes

Analyses examine pregnancy outcomes following a contraceptive failure. A contraceptive failure that results in a pregnancy can end in a live birth or a termination (stillbirth, induced abortion, miscarriage). A logistic regression was used where the outcome variable was pregnancy outcome (birth or termination) and the explanatory variable was the contraceptive method that failed. The resulting odds ratio measures the odds of a pregnancy ending in a termination – rather than a live birth – by the method that failed.

### Methods and data summary

This analysis considers failure, discontinuation, and switching of contraceptive methods as well as pregnancy outcomes. It aims to:

1. Use multiple-decrement life tables to measure:
  - Contraceptive discontinuation by reason for discontinuation for individual methods at 12, 24, 36 and 48 months
  - Contraceptive failure for individual methods at 12, 24, 36 and 48 months
  - Switching for individual methods at 12, 24, 36 and 48 months
2. Use event history analysis to measure:
  - Contraceptive failure by groups of methods based on Festin et al’s (YEAR) typology of effectiveness
  - Discontinuation with the methods group based on self vs. provider-discontinued methods
  - Switching to individual methods
  - Sociodemographic characteristics (education, wealth index, urban vs. rural, marital status, age, ethnicity and parity) and their association with failure, discontinuation and switching
3. Measure pregnancy outcomes, meaning whether it ends in birth or termination, following a contraceptive failure

Individuals were included in the analysis if they reported using at least one contraceptive method for at least one month at any point in the calendar. The final sample size of the analysis is 8,334 individuals. Data analysis for both methods was conducted in StataSE 18.^58^

### Statement of positionality

Any research undertaken is shaped by the researcher’s positionality, which requires recognition and reflexivity by the researcher. I am a PhD student based in the United Kingdom. I am a Black African woman (she/her) who has lived and worked in different countries and contexts, including where the data used in this study was collected. I work with mixed methods and have a background in demography, global health and economics. My position as a foreigner who has spent considerable time living in Global Minority countries, amidst other privileges, means that I cannot understand and connect to certain aspects of respondents’ lived experiences that are analysed within this paper. Despite these limitations in connection and understanding, I did my best to be sensitive to the nuances that exist in the data, specifically, and in respondents’ lived experiences, more broadly. In light of my positionality and my belief in the need for evidence-based decision-making, I aim to disseminate my research not only in academic spaces but also in ways that are accessible and useful to practitioners, policymakers, advocacy groups and communities where the data were collected, with the hope that the findings can contribute to ongoing discussions on contraceptive access and reproductive autonomy.

### Data quality challenges in measuring failure

There were notable discrepancies when calculating contraceptive failure using respondents’ self-reported reasons for discontinuation versus identifying when a pregnancy immediately succeeded a month where contraceptive use was reported. 303 respondents reported “became pregnant while using” as the reason for contraceptive discontinuation. When accounting for a pregnancy being reported the month after use of a contraceptive was reported – not accounting for self-reported reason for discontinuation – there were 827 instances when a pregnancy immediately followed a month of contraceptive use (following truncation and censoring, this number decreased to 596). In most of the 827 cases, the self-reported reason was “desire to become pregnant” rather than “became pregnant while using”. Contraceptive failure is defined as the proportion of contraceptive use that resulted in a pregnancy or a pregnancy termination in the month directly following a month of contraceptive use. A novel failure code was generated – the derived failure code – that relied on the raw data to measure “became pregnant while using” alongside using self-reported failure. The difference in contraceptive failure rates using the self-reported reason for discontinuation and the derived failure code is notable (Table 2). This study presents findings using both the self-reported failure and the derived failure code. The derived failure code is not intended to override respondents’ self-reports but rather relies solely on the calendar to measure failure. The contraceptive failure rate likely lies somewhere between the rate calculated using self-reported failure and the rate calculated using the derived failure code.

**Table 2.** Number of observations of failures, discontinuation by self-reported reason and switching (2017–2022)

Method	Method failure (self-reported) (%)	Method failure (derived failure code) (%)	Wanted to become pregnant (%)	Fertility and marital-related reasons (%)	Side effects/health concerns (%)	Wanted more effective method (%)	Method and accessibility barriers (%)	Other/DK (%)	Missing (%)	Switching (%)
***Pill***	48 (23.4)	94 (16.5)	151 (17.6)	127 (18.6)	152 (14.1)	165 (26.0)	96 (22.4)	4 (7.3)	26 (28.6)	189 (20.8)
***IUD***	1 (0.5)	16 (2.8)	32 (3.7)	0 (0)	30 (2.8)	2 (0.3)	7 (1.6)	0 (0)	2 (2.2)	13 (1.4)
***Injectable***	62 (30.2)	212 (37.2)	367 (42.9)	273 (40)	483 (44.7)	195 (30.7)	234 (54.7)	16 (29.1)	30 (33.0)	260 (28.6)
***Condoms***	15 (7.3)	30 (5.3)	46 (5.4)	104 (15.2)	17 (1.6)	64 (10.1)	19 (4.4)	5 (9.1)	6 (6.6)	56 (6.2)
***Periodic abstinence***	38 (18.5)	67 (11.8)	49 (5.7)	55 (8.1)	0 (0)	61 (9.6)	7 (1.6)	2 (3.6)	11 (12.1)	42 (4.6)
***Withdrawal***	16 (7.8)	28 (4.9)	11 (1.3)	21 (3.1)	1 (0.1)	38 (6.0)	6 (1.4)	4 (7.3)	5 (5.5)	33 (3.6)
***Implant***	20 (9.8)	110 (19.3)	191 (22.3)	67 (9.8)	373 (34.5)	88 (13.9)	44 (10.3)	3 (5.5)	7 (7.7)	161 (17.7)
***Emergency contraceptive***	5 (2.4)	13 (2.3)	9 (1.1)	36 (5.3)	24 (2.2)	22 (3.5)	15 (3.5)	2 (3.6)	4 (4.4)	15 (1.7)
***Total***	205 (100)	570 (100)	856 (100)	683 (100)	1080 (100)	635 (100)	428 (100)	55 (100)	91 (100)	909 (100)

## Results

Side effects are the most common reason for discontinuing, followed by a desire to become pregnant (Table 2). Though method and accessibility barriers are one of the lowest reported reasons for discontinuing, this reason predominantly affects injectable users. The implant, pill, injectable and periodic abstinence had the highest number of self-reported method failures (Table 3). In most discontinuation categories, injectable and IUD users were most likely to discontinue; these method users, along with pill users, are the most likely to switch (Table 3).

**Table 3.** Discontinuation, failure, and switching by method (per 100 episodes of use) and duration of use in Kenya (DHS data 2017–2022)

Reasons for Discontinuation																				
Method	Method failure (self-reported)				Method failure (derived failure code)				Desire to become pregnant (self-reported)				Desire to become pregnant (derived failure code)				Side effects/ other health concerns			
	12 Months	24 Months	36 Months	48 Months	12 Months	24 Months	36 Months	48 Months	12 Months	24 Months	36 Months	48 Months	12 Months	24 Months	36 Months	48 Months	12 Months	24 Months	36 Months	48 Months
***All methods***	1.5	2.6	2.9	3.2	3.3	6	7.7	8.9	5.5	10.3	14.7	175	4.6	8.2	11.6	13.8	7.9	11.3	14.4	16.0
***Emergency contraceptive***	0.8	1.5	1.5	1.5	5.9	7.5	7.5	8.7	7.6	8.5	8.5	8.5	3.4	3.4	3.4	3.4	16	16.1	16.1	16.1
***Implant***	0.4	0.9	1	1.2	1.6	4.1	6.1	7.0	2.3	7.4	14.1	16.7	1.7	5.6	11.2	13.4	6.1	11.9	18.5	21.1
***Injectable***	1.5	1.9	2.1	2.1	3.5	5.3	6.7	7.6	7.5	12.5	16	19.1	6.7	10.9	13.5	15.9	11.7	15.5	18.3	19.7
***IUD***	0	0	0	1.6	0.8	6.4	8.2	12.3	3.4	12.7	17	26.1	2.9	6.9	9.3	15.9	4.6	8.5	11.6	14.1
***Condoms***	1.3	3.6	3.8	5.2	3	5.8	9.4	10.8	4.2	8.8	14.3	14.3	3.7	7.9	13.2	13.2	2.5	3.0	3.9	3.9
***Periodic abstinence***	5.7	8.7	10.1	11	8.7	12.9	15.7	17.2	6.6	11.9	16.6	17.2	4.9	9.1	12.3	12.3	0	0	0	0
***Pill***	2.5	3.9	4.9	5.6	4.8	7.3	8.6	9.7	9.1	13	16.2	19.4	7.6	10.5	13.3	16.1	12.3	13.5	14.8	16.4
***Withdrawal***	2.8	12	12.2	12.2	3.6	15.2	15.4	18.9	3.2	6.3	7.6	11.1	2.6	3.4	4.8	4.8	0	0	1.3	1.3
																				
Reasons for discontinuation																				
Method	Fertility and marital related reasons				Wanted more effective method				Method and accessibility reasons				Other/ DK				Switching			
*** ***	12 Months	24 Months	36 Months	48 Months	12 Months	24 Months	36 Months	48 Months	12 Months	24 Months	36 Months	48 Months	12 Months	24 Months	36 Months	48 Months	12 Months	24 Months	36 Months	48 Months
***All methods***	5.2	6.24	7.5	9.0	6.2	7.8	9.2	10.4	2.3	3.1	3.8	4.3	5.1	6.6	8.2	9.4	7.8	10	12.2	14.2
***Emergency contraceptive***	24.4	24.4	24.4	27.0	10.9	10.9	10.9	10.9	6.4	6.4	6.4	6.4	4.2	4.2	8.4	8.4	9.1	9.1	9.1	9.1
***Implant***	0.4	0.9	1.7	2.1	0.8	1.5	4.2	6.6	0.4	0.5	1.6	1.7	2.6	4.2	6.4	8.4	2.5	4.4	9.3	12.5
***Injectable***	4.7	6.6	8.2	10.5	4	5.9	6.6	7.3	2.8	4.5	5.3	5.7	8.5	10.4	12.3	13.8	7.4	10.3	11.4	12.4
***IUD***	0	0	0	0	0	0.1	0.1	0.1	0.5	0.5	0.5	1.4	2.4	2.6	3.5	3.5	2.8	2.8	4.4	5.2
***Condoms***	13.1	15	17	21.5	5.7	8.4	10	10.3	1.6	2.3	3.5	3.5	3.4	3.7	4.1	4.1	6.5	9	10.5	11.2
***Periodic abstinence***	5.8	8	9.1	11.0	8.2	10.6	13.2	14.4	0.4	0.7	0.7	0.7	3.1	3.5	3.8	3.8	5.4	7.1	8.7	8.9
***Pill***	8.7	9.7	10.1	11.3	11	12.6	13.9	15.1	6.4	7	7.2	8.7	4.8	6.4	6.9	7.0	14.4	15.8	17.2	21.1
***Withdrawal***	7.1	12.7	14.3	14.3	18.6	23.5	24.8	24.8	1.7	2.4	2.6	2.6	4	8.4	9.8	12.2	17.8	25.3	26.8	26.8

### Reason for discontinuation: self-report vs. derived failure code

There is a notable difference in the self-reported and derived measures of failure (Table 3). Using the self-reported value, the IUD does not fail from 12 to 36 months; however, using the derived failure code, the IUD fails at 8.2 per 100 episodes of use at 36 months. At 36 months, estimates using individual self-reported reasons ranged from a third of the failure rate using the derived failure variable (e.g. the injectable) to a fivefold increase for the emergency contraceptive and a sixfold increase for the implant.

### Failure

Multiple-decrement life table analysis (Table 3) shows that all methods have a higher likelihood of failing as their duration of use increases. Using self-reported failure, LARCs have the lowest failure rate across all time periods, followed by the emergency contraceptive pill, injectable and then short-acting methods. Using the derived failure code, at 12 months, LARCs have the lowest likelihood of failing. The male condom, injectable and withdrawal have similar failure rates at 12 months. Using the derived failure code by 36 months, the IUD’s likelihood of failing is comparable to the pill, whereas using the self-reported failure, the pill fails approximately five times more than the IUD. Methods like the emergency contraceptive pill do not have sharp increases in failure over time; the likelihood of failing at 12 months is 5.9 per 100 episodes of use compared to 8.7 per 100 episodes of use at 48-months (derived failure). In contrast, the IUD changes from 0.8 per 100 episodes of use at 12 months, the lowest failure rate among all methods, to 12.3 per 100 episodes of use at 48 months – the highest failure rate among modern methods at this duration when using the derived failure code. There does not appear to be a duration where failure sharply increases; rather, failure increases over time for methods, albeit at different increments.

**Figure 2.**
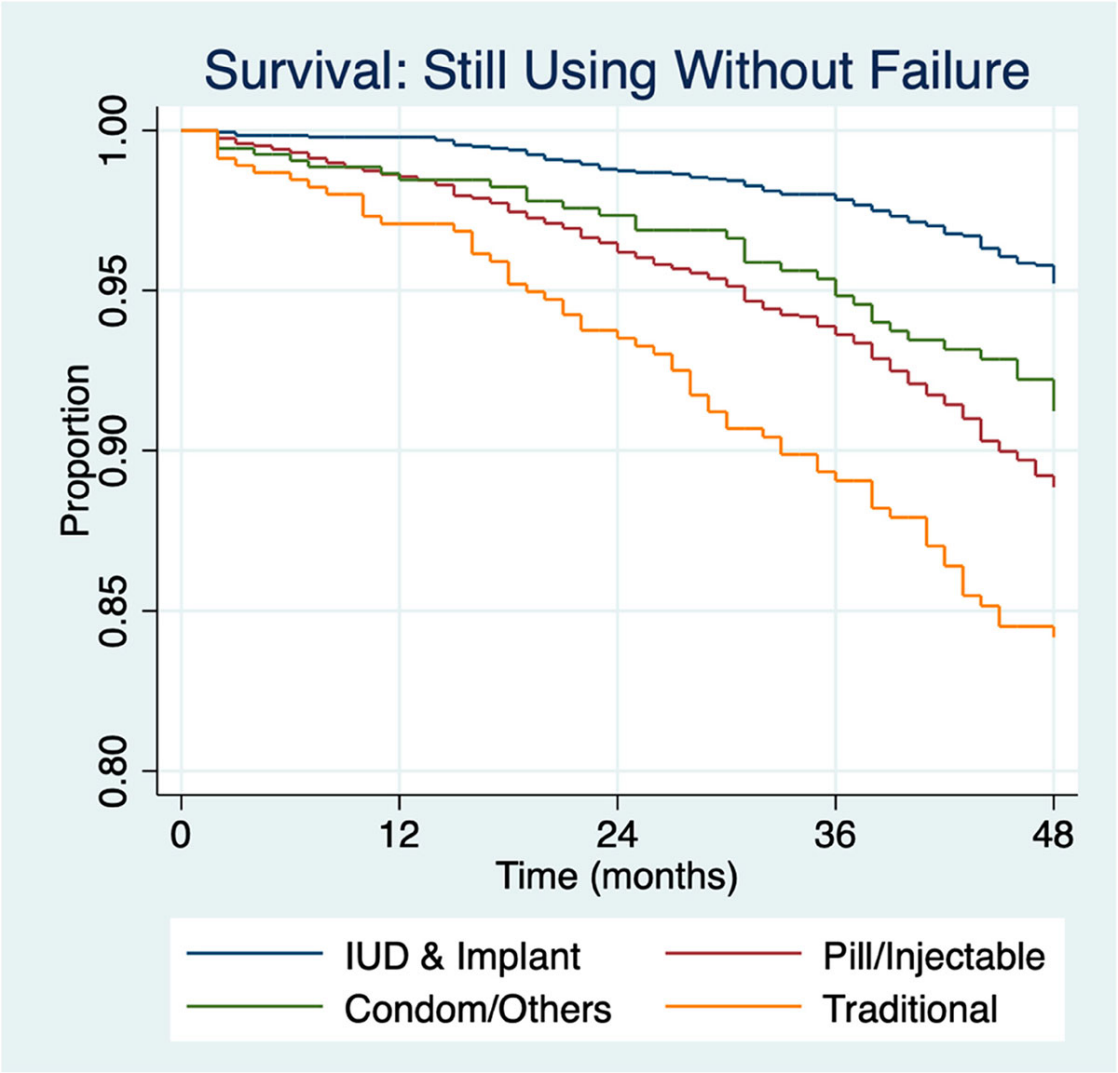
Survival: still using without failure over time and proportion of users. Kaplan–Meier survival estimates of continued contraceptive use without failure by method group. Survival probabilities are unadjusted

EHA shows that across all time periods, LARCs are the most effective contraceptive group; however, there is an increased odds of failure over time (Figure 2). At 12 months LARC users have 93% lower odds of pregnancy compared to traditional method users. By 48 months, LARC users have 77% lower odds of pregnancy compared to the reference category. Both more effective and less effective short-acting methods have higher odds of failure compared to LARCs but are less likely to fail compared to traditional methods.

### Discontinuation

Several methods (IUD, injectable, implant) show increases in discontinuation due to reported side effects over time (Table 3); the implant increased by 15.1 per 100 episodes of use from 12 months to 48 months. Other methods have less stark increases over time. The largest increases in discontinuation due to side effects tend to occur between 12 and 24 months. Users of traditional methods, like periodic abstinence, do not report discontinuation due to side effects.

Discontinuation due to a desire to become pregnant is the second most common reason for discontinuation. When using both measures of failure, LARCs are among the methods least likely to be discontinued at 12 months due to the desire to get pregnant (Table 3). In contrast, injectable discontinuation at 12 months is 7.5 per 100 episodes of use using the self-report and 6.7 for derived failure. LARCs experience an increase in discontinuation for this reason, and by 48 months, their discontinuation is nine times higher (implant) and five times higher (IUD) than at 12 months using the derived failure code. Discontinuation is more marked using the self-report; by 48 months 16.7 implant users per 100 episodes of use and 26.1 IUD users per 100 episodes of use are discontinuing due to a desire to become pregnant. Other short-acting methods like the pill experience less stark discontinuation rates over time due to a desire to get pregnant, with a two-fold increase between 12 and 48 months (derived code).

A few IUD users reported wanting a more effective method (Table 3). Traditional method users and users of more effective short-acting methods are more likely to cite desire for a more effective method as their reason for discontinuation over time. However, implant users increasingly cite wanting a more effective method over time (0.8 per 100 episodes of use at 12 months vs. 6.6. per 100 episodes of use at 48 months).

**Figure 3.**
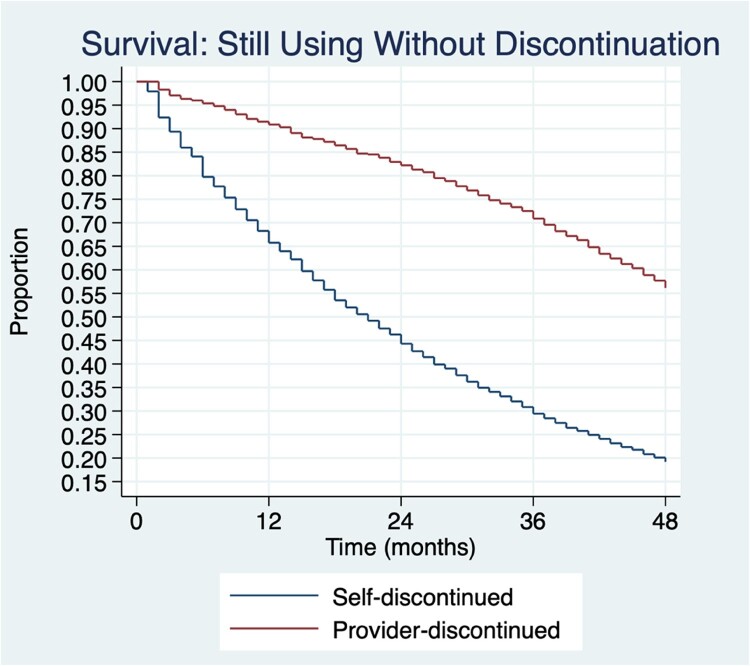
Survival: still using without discontinuing over time and proportion of users Kaplan-Meier survival estimates of continued contraceptive use without discontinuing by method discontinuation type. Survival probabilities are unadjusted.

Across all time periods, users of provider-discontinued methods are less likely to discontinue than users who can self-discontinue (Figure 3). At 12 months, provider-discontinued methods are associated with 77% lower odds of discontinuation compared to self-discontinued method users. This changes to 76%, 71% and 63% lower odds of discontinuation at 24, 36 and 48 months, respectively.

### Switching

In the life tables, across all time periods, IUD users are least likely to switch (Table 3). Despite also being a provider-discontinued method, switching from the implant increases from 2.5 per 100 episodes of use at 12 months to 12.5 per 100 episodes of use at 48 months. Pill and withdrawal users are the most likely to switch over time.

In EHA, almost all contraceptive users are more likely to switch when compared to injectable users (Table 4). The only exception is LARC users who are less likely to switch across all time periods compared to injectable users.

**Table 4.** Regression results for switching to another method

Method	12-month OR (95% CI)	12-month *p*-value	24-month OR (95% CI)	24-month *p*-value	36-month OR (95% CI)	36-month *p*-value	48-month OR (95% CI)	48-month *p*-value
Injectable	1							
Pill	5.39 (3.07–9.46)	<0.001	2.71 (2.04–3.60)	<0.001	2.42 (1.93–3.04)	<0.001	2.21 (1.81–2.71)	<0.001
IUD	0.465 (0.061–3.49)	0.457	0.317 (0.115–0.869)	0.026	0.226 (0.99–0.514)	<0.001	0.257 (0.136–0.487)	<0.001
Condoms	5.26 (2.51–11.0)	<0.001	1.69 (1.05–2.72)	0.031	1.21 (0.815–1.80)	0.343	1.04 (0.740–1.48)	0.791
Emergency contraceptive	3.79 (1.07–13.5)	0.40	2.91 (1.32–6.38)	0.008	2.16 (1.03–4.52)	0.042	2.10 (1.09–4.06)	0.027
Periodic abstinence	1.37 (0.260–4.13)	0.566	0.978 (0.565–1.69)	0.938	0.765 (0.488–1.20)	0.246	0.707 (0.477–1.05)	0.085
Withdrawal	9.69 (3.99–23.5)	<0.001	2.71 (1.39–5.25)	0.003	2.28 (1.36–3.81)	0.002	2.49 (1.62–3.82)	<0.001
Other modern methods	21.7 (2.02–231)	0.001	3.93 (0.459–33.7)	0.211	2.06 (0.229–18.6)	0.518	1.59 (0.175–14.5)	0.680
Implant	0.639 (0.296–1.38)	0.257	0.356 (0.237–0.532)	<0.001	0.418 (0.319–0.545)	<0.001	0.478 (0.386–0.593)	<0.001
Standard days	1.77 (0.220–14.3)	0.590	0.364 (0.051–2.62)	0.315	0.217 (0.031–1.51)	0.123	0.151 (0.022–1.05)	0.056

OR =  odds ratio; CI = confidence interval. Table 4 results show the unadjusted logistic regression model with contraceptive method as the sole predictor of switching.

**Figure 4.**
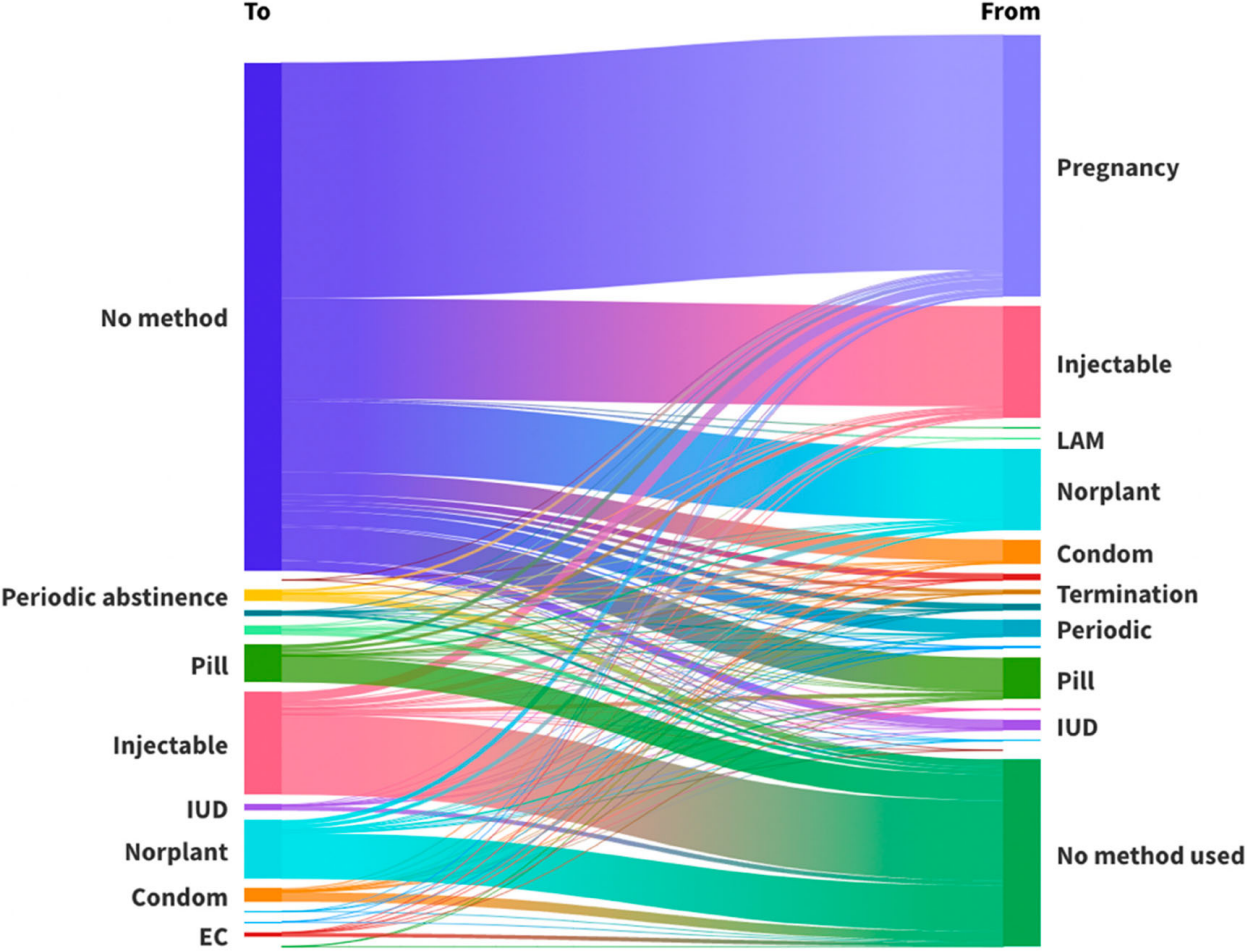
Contraceptive switching, excluding births and terminations

Users tend to switch from no method (left-hand side) to pregnancy, the injectable or the implant (right-hand side) (Figure 4). A notable proportion of current users (right-hand side) switch to no method (left-hand side), likely due to changes in fertility intentions and a desire to become pregnant, although this cannot be measured as calendar data does not include fertility intentions. Figure 4 does not consider time and includes multiple switches by an individual.

### Sociodemographic characteristics

Compared to the reference category of 25–34 year olds, younger (≤24 years) women are more likely to experience failure, discontinue and switch methods and older (≥35 years) women are less likely than the reference category (Table 5). Married/cohabitating and widowed/divorced women are more likely to discontinue and switch than those never in union. Compared to Kikuyu women, Somali women (who make up less than 2% of the sample population) are three times more likely to experience failure and twice as likely to discontinue. Wealthier income groups are less likely to experience failure and discontinue but are more likely to switch compared to the poorest income group. The likelihood of a method failing or discontinuing a method increases as parity increases; women with 3 + births are 16 times more likely to experience failure (OR: 16.4, CI: 7.24–37.04) and twice as likely to discontinue (OR: 2.13, CI: 1.64–2.64). However, it should be noted that the confidence interval on the former is quite large. The association between switching and parity follows a similar trend, with those who have given birth more likely to switch than nulliparous women.

**Table 5.** Sociodemographic characteristics and failure, discontinuation and switching (2017–2022)

Covariate	Failure OR (95% confidence interval)	Failure *p*-value	Discontinuation OR (95% confidence interval)	Discontinuation *p*-value	Switching OR (95% confidence interval)	Switching *p*-value
**Method**						
Least effective (withdrawal, periodic abstinence, other traditional methods)	1					
LARCs	0.209 (0.157–0.276)	<0.001	0.485 (0.427–0.550)	<0.001	0.430 (0.331–0.563)	<0.001
More effective short-acting (pill, injectable)	0.514 (0.399–0.661)	<0.001	1.33 (1.18–1.50)	<0.001	1.14 (0.892–1.46)	0.250
Less effective short-acting (condoms, emergency contraception, standard days, other modern methods)	0.803 (0.552–1.17)	0.251	1.343 (1.13–1.58)	0.001	1.34 (0.968–1.89)	0.076
**Urban vs. rural**						
Urban	1					
Rural	1.07 (0.836–1.38)	0.566	1.05 (0.956–1.15)	0.325	1.20 (0.994–1.45)	0.058
**Age**						
25–34	1					
15–19	3.19 (1.88–5.40)	<0.001	1.49 (1.22–1.82)	<0.001	1.49 (0.927–2.38)	0.099
20–24	1.75 (1.38–2.20)	<0.001	1.42 (1.29–1.55)	<0.001	1.50 (1.25–1.81)	<0.001
35–44	0.452 (0.355–0.576)	<0.001	0.670 (0.615–0.729)	<0.001	0.777 (0.642–0.939)	0.009
44+	0.135 (0.060–0.303)	<0.001	0.571 (0.476–0.684)	<0.001	0.640 (0.430–0.952)	0.028
**Marital status**						
Never in union	1					
Married/cohabitating	1.44 (0.922–2.24)	0.108	1.35 (1.16–1.56)	<0.001	2.31 (1.62–3.28)	<0.001
Widowed/divorced/separated	0.961 (0.571–1.61)	0.880	1.34 (1.13–1.59)	0.001	1.66 (1.12–2.47)	0.012
**Ethnicity**						
Kikuyu	1					
Embu	1.02 (0.448–2.29)	0.973	0.850 (0.651–1.11)	0.233	1.18 (0.757–1.84)	0.462
Kalenjin	.909 (0.674 - 1.22)	0.529	0.909 (0.816–1.01)	0.085	0.824 (0.661–1.02)	0.087
Kamba	1.08 (0.776–1.48)	0.663	0.825 (0.729–0.993)	0.002	0.775 (0.592–1.02)	0.064
Kisii	0.749 (0.491–1.42)	0.180	0.771 (0.662–0.898)	0.001	0.563 (0.404–0.784)	0.001
Luhya	0.961 (0.696–1.32)	0.811	1.01 (0.910–1.14)	0.721	0.731 (0.571–0.937)	0.013
Luo	0.928 (0.678–1.27)	0.644	0.999 (0.901–1.13)	0.882	0.638 (0.501–0.813)	<0.001
Maasai	1.65 (1.08–2.52)	0.020	0.884 (0.715–1.09)	0.256	0.703 (0.438–1.12)	0.143
Meru	0.930 (0.641–1.35)	0.702	1.08 (0.935–1.24)	0.297	1.04 (0.784–1.39)	0.763
Mijikenda/Swahili	0.429 (0.227–0.808)	0.009	1.02 (0.864–1.21)	0.786	0.792 (0.537–1.17)	0.241
Somali	3.21 (1.51–6.83)	0.002	2.04 (1.51–2.75)	<0.001	0.192 (0.028–1.28)	0.089
Taita/Taveta	0.414 (0.114–1.51)	0.181	0.935 (0.786–1.51)	0.600	1.04 (0.608–1.78)	0.882
Other	1		0.944 (0.512–1.71)	0.828	0.542 (0.085–3.46)	0.517
**Education**						
No primary	1					
Primary	1.02 (0.660–1.59)	0.914	0.824 (0.710–0.958)	0.012	1.26 (0.786–2.03)	0.333
Secondary	1.19 (0.753–1.87)	0.456	0.910 (0.778–1.06)	0.242	1.36 (0.845–2.21)	0.202
Higher	1.53 (0.957–2.47)	0.075	0.971 (0.819–1.15)	0.737	1.48 (0.907–2.43)	0.116
**Income**						
Poorest	1					
Poor	0.789 (0.590–1.06)	0.112	0.911 (0.815–1.01)	0.072	1.14 (0.875–1.48)	0.328
Middle	0.794 (0.596–1.06)	0.114	0.915 (0.812–1.01)	0.065	1.28 (0.989–1.65)	0.060
Richer	0.701 (0.507–0.969)	0.032	0.916 (0.795–1.02)	0.101	1.10 (0.829–1.48)	0.484
Richest	0.833 (0.564–1.23)	0.360	0.988 (0.835–1.12)	0.692	1.48 (1.05–2.07)	0.024
**Parity**						
0	1					
1	5.57 (2.62–11.8)	<0.001	1.75 (1.41–2.17)	<0.001	2.61 (1.66–4.11)	<0.001
2	10.2 (4.63–22.6)	<0.001	1.97 (1.52–2.40)	<0.001	2.47 (1.51–4.03)	<0.001
3+	16.4 (7.24–37.0)	<0.001	2.13 (1.64–2.64)	<0.001	2.37 (1.44–3.90)	0.001

OR = odds ratio. CI = confidence interval. Results from the logistic regression model are adjusted for contraceptive method type, place of residence, age, marital status, ethnicity, educational attainment, income and parity.

### Pregnancy outcome

Pregnancy outcome – whether a pregnancy ended in a birth or termination – among respondents whose contraceptive method failed was measured (Table 6). There are a total of 524 contraceptive failures that included the pregnancy outcome in the calendar, since some respondents’ calendars ended when they were still pregnant. More effective short-acting method users are 54.1% less likely to have a termination compared to IUD and implant users who experienced a contraceptive failure. Less effective short-acting method users are 40.2% less likely, and traditional method users 65.4% less likely when compared to implant and IUD users.

**Table 6.** Odds of pregnancy termination (vs. live birth) by method of contraceptive failure

Method	OR	*p*-value
IUD and implant	1	
Pill, injectable	0.472 (0.238–0.935)	0.031
Condoms, emergency, standard days, other modern methods	0.598 (0.188–1.89)	0.382
Withdrawal, abstinence, other traditional methods	0.345 (0.121–0.983)	0.046

OR = odds ratio. CI = confidence interval. Results are unadjusted logistic regression with contraceptive method that failed as the sole predictor of pregnancy outcome (termination vs. live birth).

## Discussion

This paper measures contraceptive failure using self-reported reasons for discontinuation and a derived failure code that identifies a contraceptive failure when a pregnancy is reported in the month immediately succeeding use of a contraceptive method in the data. This analysis suggests that when using the derived failure code in Kenya, at certain durations, LARCs have a failure rate comparable to short-acting methods, and the likelihood of a LARC failing increases with duration of use. This higher LARC failure rate contrasts with findings from other clinical and typical-use studies. Clinical studies often have a 100% efficacy rate (Table 1). Polis et al^30^ found failure rates as high as 2.2 per 100 episodes of use for the IUD and 1.3 per 100 episodes of use for the implant. At 12 months, this study found a failure rate of 1.6 per 100 episodes of use for the implant and 0.8 per 100 episodes of use for the IUD, while at 48 months, these increased to 7.0 and 12.3 per 100 episodes of use, respectively. Both LARCs and short-acting methods are much more effective than traditional methods. Alongside using self-reported failure, the derived failure code uses the calendar data as a sensitivity to calculate typical-use failure. True typical-use failure likely lies within the range of the self-reported and the derived failure code.

Understanding the effectiveness of methods and communicating their risks and benefits clearly is crucial for contraceptive counselling to ensure a potential user is knowledgeable about each method and can make an informed choice about what works best for them. Follow-up care can also support users to manage side effects, ensure correct use, and aid in discontinuation and/or switching that better supports an individual’s reproductive goals. It is also important to have a healthcare system that can support individuals who experience contraceptive failure, ensuring safe abortion services and maternal health services are accessible.

Studies that assess typical-use failure tend to measure contraceptive failure in 12 month increments,^49,59–61^‡ less often up to 24 and/or 36 months.^27,30^ Moreau et al^48^ measured failure up to 60 months; however, the implant was not included in their analysis. This paper measured failure up to 48 months for all methods available in the dataset, finding that the failure rate for all methods increased between 36 and 48 months. Given the nature of long-acting methods, longer time periods for assessing failure, discontinuation, and switching are essential to capture the longer periods of (intended) use for LARCs.

This work highlights concerns in the calculation of contraceptive failure due to an under- or misreporting of failure. Using the derived versus self-reported failure increased failure rates. Calendar data has been used to measure typical-use failure, specifically in Global Majority countries where longitudinal and/or other sources of calendar data are not always readily available.^30,49^ In this study, there were 365 more contraceptive failures in the derived failure code than in the self-reported. In Tumlinson & Curtis,^62^ 38% of women who reported a contraceptive failure in the first wave of their panel data then reported a “desire to get pregnant” in the second wave two years later, highlighting how reports of contraceptive failure can change over time. Reproductive histories in the form of a calendar rather than asking about failure specifically allow for discrepancies to be identified, but other datasets using retrospective self-reported failure may also be underestimating failure. Such discrepancies can impact our understanding of contraceptive failure.

A potential explanation for this discrepancy is the tendency for individuals to change their reporting of a pregnancy’s intendedness from pre-birth to post-birth, for example, an initially reported unintended pregnancy subsequently reported as intended. Studies have found that some women who report unwanted/unintended pregnancies later change their report to wanted/intended following birth.^63–66^ Chamberlain et al^67^ interrogated how relationships, parenting, and the economic situation between pre- and post-birth changed how respondents recalled their pregnancy intendedness. The discrepancy detected in the reporting of contraceptive failure in this paper may be a quantitative manifestation of how recollection bias influences data quality, especially as it relates to contraceptive failure and the recollection of pregnancy intendedness. Social desirability bias likely also plays a role, as women may be reluctant to describe their child as unintended, unwanted or mistimed and may report the child as wanted post-birth.^68,69^ The majority of contraceptive failures reported by respondents as “desire to become pregnant” rather than “became pregnant while using” suggests that respondents may be recalling their pregnancy intendedness differently following birth.

LARC users in Kenya are less likely to discontinue use when compared to other methods. This analysis is unable to determine why this may be the case; speculatively, it may be due to users selecting these methods due to their long-acting abilities and desire to avoid pregnancy, these methods being selected for their higher efficacy, and/or due to barriers experienced when removal is desired. For LARC users who discontinue, the common self-reported reasons for discontinuation (Tables 2 and 3) are side effects health concerns, desire to become pregnant, and, when using the derived failure code, contraceptive failure. Recent literature has found that methods may be discontinued or switched from due to the impact it has on pleasure and an individual’s sex life.^29^ This is not a reason available in DHS calendar data; future research should explore the applicability of this to contraceptive dynamics in this context and/or specifically for LARC users. Research elsewhere, including in Africa, has found that users experience barriers in having their LARCs removed.^12,14,15,20,23^. This includes providers refusing to remove methods, pressuring users to use methods for their full duration despite side effects or desire to become pregnant and high removal fees.^16,70,71^ Providers can play a gatekeeping role in deciding legitimate reasons to discontinue use.^16^ Facility readiness and having access to the human and material resources needed for LARC removal can impede LARC removal for users who want to discontinue.^72,73^ Brunie et al^71^ found that some respondents who had not seen a provider but expressed a desire for discontinuation reported needing to find the right time to visit the facility, waiting for their next regular appointment, and saving money to afford removal fees. A provider-discontinued method is susceptible to provider and facility-level constraints as well as other individual-level barriers in accessing removal services, including time and financial barriers. Future studies should explore why LARC users in Kenya are less likely to discontinue and switch when compared to other contraceptive method users.

Findings also showed that some implant users discontinue due to a desire for a more effective method. Considering implants are one of the most effective methods available, their increased discontinuation due to this reason was a surprising finding. This may either signal a lack of understanding of method effectiveness, which can be improved through contraceptive counselling, or can be the result of users transitioning from LARCs to sterilisation – which appears to only affect implant and not IUD users. However, sterilisation rates in Kenya are very low, with 1% of sexually active unmarried women and 2% of currently married women reporting being sterilised in the Kenya 2022 DHS.

Compared to injectable users, across all time periods, LARC users are less likely to switch, the only method for which this is the case. LARC users may be less likely to switch due to satisfaction with their method, the long-acting nature of LARCs or barriers to removal, which reduce the ability to switch. Though this analysis cannot include fertility intentions, LARC users may be switching to methods that better align with dynamic fertility intentions. A US study found that method satisfaction and confidence influenced switching intentions with IUD users, relative to other contraceptive method users, being more likely to have very low switching intentions.^73^ Future research should explore the relationship between switching, fertility intentions and other contraceptive perceptions, with a particular focus on what LARC users are switching to and why.

Some sociodemographic characteristics have an influence on experiences of failure, discontinuation and switching. This study found that younger age groups (≤24 years) are more likely to experience failure and discontinuation, and older age groups (≥35 years) are less likely. This finding is aligned with similar studies. Polis et al^30^ found that women under 25 had higher failure rates (using self-reported data) than those in older age categories for every method except the implant. Using data from 15 DHS, Bradley et al^74^ found that failure disproportionately affects the youngest and poorest women. Ontiri et al^75^ found that discontinuation was more likely among short-acting users and younger women (ages 15–19 and 20–24). The likelihood of experiencing failure and discontinuation was found to increase as parity increased. These findings contrast with those of Polis et al^30^ who found that 12-month failure rates were higher among lower parity women. Mekonnen & Wubneh^76^ found that not having any children was associated with contraceptive discontinuation. However, the findings of Sundaram et al^47^ are similar to those in this analysis; in their assessment of 12-month failure rates, they found the probability of failure increased from 5% among users with no children to 14% for those with one child and 15% among the group with 2 + children. This study also found the Somali women – who made up only a small proportion (2%) of the sample population – were more likely to discontinue and experience failure. Future qualitative research should focus on the contraceptive dynamics of different ethnic and social groups as disaggregation allows for a better understanding of the unique experiences of certain groups and identifies how they can be better supported in their contraceptive use and care.

Over time, LARC users are more likely to cite their reason for discontinuing as a desire to become pregnant. Using a method of more than three years’ duration when a user potentially intends to have children in the next year or two may highlight issues related to knowledge of these methods’ long-acting nature, deficiencies in contraceptive counselling, gaps in method availability, and/or coercion during counselling. Users may also be exercising their reproductive autonomy and choose LARCs due to their highly effective nature rather than their long-acting abilities; for example, they may choose to use a LARC to ensure they do not have a child in the next 1–2 years with the intention to remove the method early. However, a study from Western Kenya found that women experienced barriers to LARC removal and that some were reluctant to use a LARC for fear they would not be able to remove it upon request.^70^

The Kenyan analysis suggests that there may be method-specific differences in pregnancy outcome – meaning whether the pregnancy ends in a birth or termination (miscarriage, abortion or stillbirth) following contraceptive failure, specifically for LARC users. It is important to highlight that abortion is underreported globally across diverse contexts due to (il)legality and stigma.^77–79^ Some respondents in the sample may not have reported pregnancies and subsequent terminations for these reasons. Since DHS surveys are conducted by interviewers, social desirability bias may influence the reporting of terminated pregnancies.^80,81^

This paper has important implications for considering bodily and contraceptive autonomy. Higher LARC typical-use failure identified in this study has salience for contraceptive counselling. Healthcare systems may need to anticipate demand for health services such as maternal healthcare and safe abortion services to support individuals who experience an unintended/ mistimed/ unwanted pregnancy. Future research should explore what may lead to LARC failures, for example, improper insertion or expulsion of LARCs.

Future research should explore why discontinuation and switching are lower among LARC users in this context to better support efforts to deliver contraceptive services that promote bodily and reproductive autonomy. As the life tables and self-reported discontinuation show, LARC users often discontinue due to a desire to become pregnant and side effects. Ensuring LARC removal services are accessible for users who desire to become pregnant is paramount to supporting individuals in achieving their reproductive goals. Related to other areas of dissatisfaction, for example, side effects, contraceptive services should be equipped to first inform potential users of these side effects and support them in their management, should they experience them. This could include managing symptoms or switching to a different method. Finally, this analysis found that certain sociodemographic groups may need more tailored programming and support.

### Strengths and limitations

This study has six limitations, which mainly relate to the data used. While the derived failure code aligns with the standard definition of contraceptive failure, it is possible that not all contraceptive failures may be “failures” in that the individual who becomes pregnant believes the pregnancy is unintended/ mistimed/ unwanted. Trussel et al^82^ found that 59% of women who experienced a contraceptive failure reported being unhappy or very unhappy, whereas 25% were happy or very happy. Cleland & Ali^49^ found that 84% of contraceptive failures were classified by the mother as unwanted or mistimed.^49^ This research therefore recognises that there is not always a direct correlation between contraceptive failure and pregnancy (un)intendedness or wantedness.

This study uses calendar data, which is susceptible to limitations, including general reliability, validity, recall bias and social desirability bias. Bradley et al^83^ found evidence of “substantial underreporting of contraceptive use” in their analysis of calendar data. Strickler et al^84^ find relatively reliable aggregate-level data, but caution that individual-level data have lower levels of reliability. Anglewicz et al.^85^ find “moderate/ substantial” reliability of calendar data in their seven (six African) country study. Studies suggest that the more complicated the reproductive history, the less reliable the data.^27,83–85^ However, they find reports from LARC users have higher reliability than short-acting and traditional users.^27,83–85^ Given that this study focuses on LARCs, the LARC findings may be more reliable than other contraceptive categories. Calendar data is also influenced by recall bias as it asks respondents to recount details for the past five years. However, studies have suggested that collecting this data in a calendar format results in more complete and consistent data.^86,87^ Some questions are more prone to underreporting, including self-reported terminations, which were included in this analysis. Social desirability bias and stigma may lead to an underreporting of terminations, particularly abortions, and bias towards underreporting contraceptive failures.^88^

The life tables analysis uses self-reported reasons for discontinuation; previous research finds that reasons for discontinuation vary in their reliability. Sarnak et al^89^ found that the most reliable data was within the most recent 12 months; this has implications for this study, which includes up to 48 months. Hence, reasons for discontinuation, particularly in later months, may not be as reliable as in earlier months. Other studies measuring the reliability of self-reported reasons for discontinuation found moderate reliability.^62,84^.

Following standard practice, the data were truncated and censored to account for methods with unknown start and/or end dates; being able to determine when a method was initiated would have resulted in more accurate findings. Calendar data does not allow for the reporting of dual method use, meaning, for example, if an individual is using both the condom and the pill, they can only report one method. It is not possible to determine what proportion of respondents may have been using more than one method. The method they chose to report would influence the findings of this study.

This paper assessed contraceptive dynamics up to 48 months. While this better accounts for long-acting methods and is a contribution to existing literature, which tends to measure shorter time periods, this approach has shortcomings, including susceptibility to recall bias and the sample size. Table 3 provides the sample size, which, in some cases, is small and reflected in the confidence intervals. As a sensitivity test, the same analyses were conducted on the 24 most recent months of data, and the results did not notably vary from those using 48 months. Calendar data do not take into consideration fertility intentions; hence, this analysis cannot account for the influence that dynamic fertility intentions have on contraceptive behaviour, especially over longer time periods.

This study also has several strengths, including the development of a novel derived contraceptive failure variable to supplement the traditional measure of contraceptive failure. With a specific focus on LARCs, this analysis sheds light on the similarities and differences between LARC users in Kenya compared to short-acting and traditional method users. The majority of studies that assess LARC users’ experiences are qualitative; this study contributes a quantitative approach using open-access data to understand users’ experiences. Finally, it quantitatively measures contraceptive behaviour at a national level up to 48 months, accounting for the long-acting nature of LARCs.

## Conclusion

As the number of LARC users grows annually and there are calls from international and national stakeholders and decision-makers to increase LARC access and use, it is important that users’ experiences are well understood to inform user-centred family planning programming.

Our study found that LARC failure in Kenya is higher than reported by clinical studies and other studies measuring typical-use failure. This finding is likely due to the use of a derived failure code to account for discrepancies in the data. This has important implications for contraceptive counselling, particularly when individuals initiate a method, and for the healthcare system should contraceptive users experience an unintended/ mistimed/ unwanted pregnancy. LARC users are less likely to switch and discontinue compared to those using methods that can be discontinued without a provider, which may indicate method satisfaction or experiencing barriers in LARC removal. Should it be the latter, as other studies have found, this analysis further emphasises the need for accessible removal services that allow individuals to make informed decisions about their body and reproduction. LARC users are also likely to report discontinuation due to a desire to get pregnant before the expiration of their method. Further research should explore whether some individuals use LARCs for their efficacy rather than their long-acting abilities. This research fills a knowledge gap by exploring LARCs in the Kenyan context and calls attention to centring users, ensuring they are well-informed and supported to make decisions about the contraceptive method(s) that will allow them to exercise their right to safe, effective contraception.

## Supplementary Material

SHRM LARC Contraceptive Dynamics Supplementary Material

## Footnotes

* FP2030 is the successor to FP2020, which ended in 2020. FP2030 is a global partnership encompassing a range of stakeholders focused on expanding access to family planning methods.

† Global Majority and Global Minority are used here to refer to the populations that make up the majority and minority of the world population, respectively. The term Global Majority includes African, Asian and Latin American countries and is used to avoid hierarchical, income-based development classifications such as “developing” and “low- and middle-income”.

‡ Mitra & Al-Sabir measure discontinuation in 24-month increments but not failure (their measurements do not include contraceptive failure)

## Provenance statement

This article was not commissioned and went through external peer review.
