# Supplementary material for: Discontinuation, switching and contraceptive failure patterns of long-acting reversible contraceptive users in Kenya: a quantitative study
Source: Sex Reprod Health Matters. 2026 Jan 9;33(1):2603740. doi: 10.1080/26410397.2025.2603740 (PMC12997378; doi:10.1080/26410397.2025.2603740)
Supplement: SHRM LARC Contraceptive Dynamics Supplementary Material [file ZRHM_A_2603740_SM8408.docx]

Regression results for failure event history analysis

| **Method Group** | **12- month OR (95% CI)** | **12-month p-value** | **24-month OR (95% CI)** | **24-month p-value** | **36-month OR (95% CI)** | **36-month p-value** | **48-months OR (95% CI)** | **48-month p-value** |
| --- | --- | --- | --- | --- | --- | --- | --- | --- |
| Withdrawal, abstinence, other traditional methods | 1 | | | | | | | |
| IUD and implant | 0.066  (0.021-0.206) | <0.001 | 0.173  (0.101-0.297) | <0.001 | 0.173  (0.114-0.263) | <0.001 | 0.232  (0.166-0.323) | <0.001 |
| Pill and injectable | 0.507  (0.270-0.949) | 0.034 | 0.592  (0.389-0.901) | 0.014 | 0.576  (0.412-0.804) | 0.001 | 0.634  (0.476-0.843) | 0.002 |
| Condoms, emergency, standard days, other modern methods | 0.572  (0.234-1.39) | 0.220 | 0.506  (0.261-0.983) | 0.044 | 0.555 (0.334-0.924) | 0.023 | 0.563 (0.369-0.858) | 0.008 |

Regression results for discontinuation event history analysis

| **MeMetho Method Group** | **12- month OR (95% CI)** | **12-month p-value** | **24-month OR (95% CI)** | **24-month p-value** | **36-month OR (95% CI)** | **36-month p-value** | **48-months OR (95% CI)** | **48-month p-value** |
| --- | --- | --- | --- | --- | --- | --- | --- | --- |
| Self-discontinued (all methods except LARCs) | 1 | | | | | | | |
| Provider-discontinued (IUD, implant) | 0.232  (0.175-0.309) | <0.001 | 0.244  (0.206-0.289) | <0.001 | 0.292  (0.260-0.328) | <0.001 | 0.367  (0.337-0.400) | <0.001 |
